# Neck sensor-supported hyoid bone movement tracking during swallowing

**DOI:** 10.1098/rsos.181982

**Published:** 2019-07-10

**Authors:** Shitong Mao, Zhenwei Zhang, Yassin Khalifa, Cara Donohue, James L Coyle, Ervin Sejdic

**Affiliations:** 1 Department of Electrical and Computer Engineering, Swanson School of Engineering, University of Pittsburgh, Pittsburgh, PA 15260, USA; 2 Department of Communication Science and Disorders, School of Health and Rehabilitation Sciences, University of Pittsburgh, Pittsburgh, PA 15260, USA; 3 Department of Bioengineering, Swanson School of Engineering, University of Pittsburgh, Pittsburgh, PA 15260, USA; 4 Department of Biomedical Informatics, School of Medicine, University of Pittsburgh, Pittsburgh, PA 15260, USA; 5 Intelligent Systems Program, School of Computing and Information, University of Pittsburgh, Pittsburgh, PA 15260, USA

**Keywords:** swallowing, hyoid bone movement, machine learning, swallowing accelerometry, dysphagia, biomedical sensor

## Abstract

Hyoid bone movement is an important physiological event during swallowing that contributes to normal swallowing function. In order to determine the adequate hyoid bone movement, clinicians conduct an X-ray videofluoroscopic swallowing study, which even though it is the gold-standard technique, has limitations such as radiation exposure and cost. Here, we demonstrated the ability to track the hyoid bone movement using a non-invasive accelerometry sensor attached to the surface of the human neck. Specifically, deep neural networks were used to mathematically describe the relationship between hyoid bone movement and sensor signals. Training and validation of the system were conducted on a dataset of 400 swallows from 114 patients. Our experiments indicated the computer-aided hyoid bone movement prediction has a promising performance when compared with human experts’ judgements, revealing that the universal pattern of the hyoid bone movement is acquirable by the highly nonlinear algorithm. Such a sensor-supported strategy offers an alternative and widely available method for online hyoid bone movement tracking without any radiation side-effects and provides a pronounced and flexible approach for identifying dysphagia and other swallowing disorders.

## 1. Introduction

Swallowing is such a natural part of our everyday experience that we often take it for granted, however, it is a complex neuromuscular process involving the coordination of physiological events in a somewhat variable sequential manner. One of the important swallow-induced events is hyoid bone movement. The hyoid bone, which is a component of the mechanism producing airway closure and oesophageal opening during swallowing, is displaced in a net upward (superior) and forward (anterior) direction reflecting the functional integrity of the suprahyoid muscles (connected with the hyoid bone) responsible for these movements [1,2]. Abnormalities in the hyoid bone movement can lead to dysphagia, or difficulty swallowing. Dysphagia may occur secondary to impairments in physiological aspects of swallowing, among which is suprahyoid muscle function. Swallowing impairments including entry of food or liquid into the airway can result in malnutrition, dehydration or aspiration pneumonia, and is often strongly associated with limited or disordered suprahyoid muscle activity and hyoid bone movement [3–7].

Videofluoroscopic swallowing study (VFSS) is one available imaging evaluation that clinicians use to evaluate airway invasion and physiological aspects of swallowing in people with dysphagia [8–10]. While VFSS provides useful images for clinicians to analyse swallow function, it is expensive, exposes patients and examiners to radiation, and is not available in institutions without X-ray departments or qualified examiners to perform and interpret the examination [11–14]. It is also not feasible in cases in which patients prefer not to undergo X-ray testing or when patients are unable to participate in the examination protocols [1,15,16]. Therefore, it is necessary to investigate alternative, non-invasive tools to evaluate swallowing by tracking the hyoid bone. The neck sensor, which collects vibratory signals, is an alternative evaluation tool that has been explored recently. Vibratory signals may be used as surrogates to imaging, tracking some physiological aspects of swallowing, such as hyoid bone movement, by placing surface sensors on the skin of a person’s anterior neck [3,17,18]. Currently, there is growing but limited research suggesting the relationship between hyoid bone movement and tri-direction vibration signals, including the anterior–posterior (A–P), superior–interior (S–I) and medial–lateral (M–L) directions [16,19–21]. In addition to this, no studies have investigated real-time tracking of hyoid bone movement using non-invasive tools, which has remained a difficult and unresolved problem for the last 20 years.

**Figure 1.**
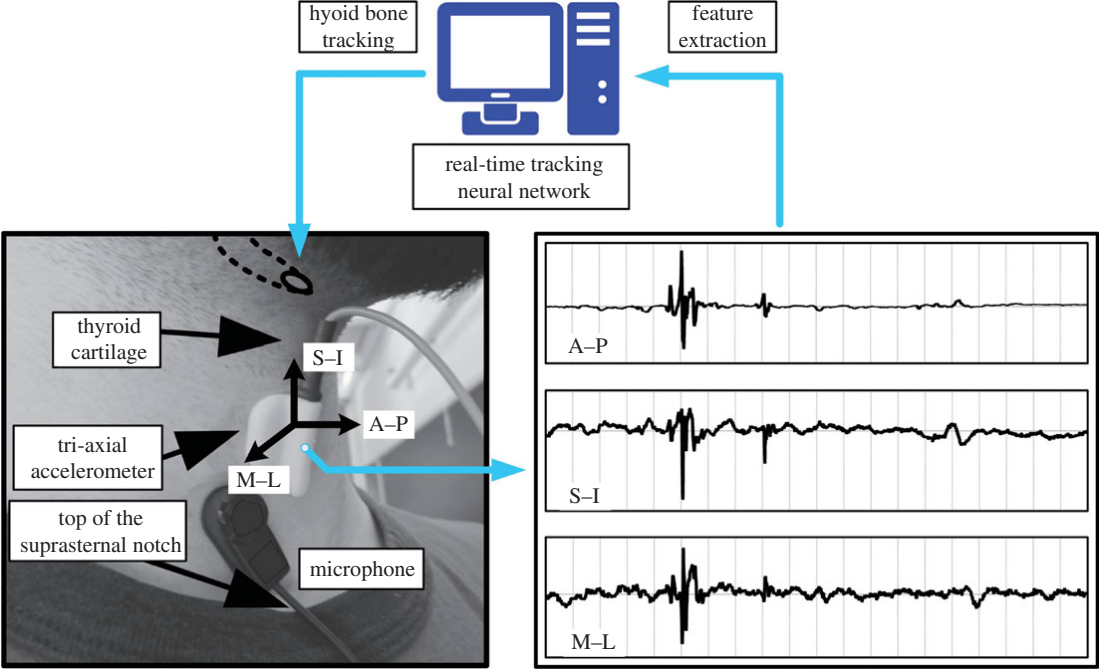
Hyoid bone tracking based on the sensor signals and dataset labelling. During a swallowing period, the neck vibration is sampled in the anterior–posterior (A–P), superior–interior (S–I) and medial–lateral (M–L) directions. In this study, the accelerometer shown in the figure is applied for all the patients. The deep learning architecture, SRNN is intended to track the hyoid bone with the informative features extracted from the multi-channel signals. The microphone shown in the figure is used for other purposes.

Despite the complexity of hyoid bone displacement with more than 30 muscles, membranes and nerves interacting, we sought to investigate the ability of tri-axial accelerometer signals to track hyoid bone movement during the pharyngeal phase of swallowing and to compare its accuracy with the gold standard of measurement: trained human judgements of hyoid bone movement using frame by frame video analysis. We hypothesized that it is feasible for a computer-aided algorithm using neck sensor signals to track hyoid bone movement (figure 1). To investigate this, we used a deep learning architecture known as stacked recurrent neural network (SRNN), which is a machine learning topology with high nonlinearity, to explore the relationship between the vibration signals and hyoid bone movement during swallowing.

## 2. Methods

### 2.1. Data collection and equipments

We collected 400 swallows from 114 enrolled patients undergoing VFSS due to suspected dysphagia at the University of Pittsburgh Medical Center Presbyterian Hospital. Participants in the study included 65 (57.02%) males and 49 females (42.98%). The median age of participants was 64 years, with a range of 19–94. Twenty-one participants (18.42%) had a history of stroke. Data were collected during VFSS as a part of routine clinical care so as not to interfere with each patient’s clinical needs as determined by the examining clinicians. As such, a speech-language pathologist conducted the VFSS and determined bolus administration order, consistencies used, bolus volume, number of trials, mode of administration of contrast, and patient position/posture and other swallow compensatory manoeuvers for patients based on clinical judgement and patient presentation of dysphagia. Consistencies used included thin (Varibar Thin Liquid with less than 5 cps consistency), nectar-thick liquid (Varibar Nectar with 300 cps consistency), pudding (Varibar Pudding with 500 cps consistency) and saliva. Bolus volume was either a self-selected comfortable volume from a cup (thin and thick liquids only, 212 swallows), 3–5 ml bolus from a spoon for all consistencies including liquids (183 swallows), or saliva (five swallows). For this study, we excluded swallows using a compensatory strategy and swallow segments containing multiple sequential swallows.

VFSSs were conducted using an X-ray machine (Precision 500D system, GE Healthcare, LLC, Waukesha, WI) and the videos were captured by a frame grabber module (AccuStream Express HD, Foresight Imaging, Chelmsford, MA) with 60 Hz sampling rate. All videos were down sampled to 30 Hz to eliminate duplicate frames.

The sensor signals were collected concurrently during all VFSS examinations using a tri-axial accelerometer neck sensor and contact microphone. The accelerometer (ADXL 327, Analog Devices, Norwood, MA) was attached at the midline of the anterior neck of participants at the level of the cricoid cartilage with double-sided tape to obtain best signal quality [22]. The sensor’s axes were aligned to the anatomical A–P, S–I and M–L directions, respectively.

The sensor was powered by a power supply (model 1504, BK Precision, Yorba Linda, CA) with a 3V output, and the resulting signals were bandpass filtered from 0.1 to 3000 Hz with 10 times amplification (model P55, Grass Technologies, Warwick, RI). The voltage signals for each axis of the accelerometry sensor were fed into a National Instruments 6210 DAQ and recorded at 20 kHz by the LabView program Signal Express (National Instruments, Austin, TX). This set-up has been shown to be effective at detecting swallowing activity in previous studies [23,24]. All the data collection protocols were approved by the University of Pittsburgh Institutional Review Board.

### 2.2. Data labelling

**Figure 2.**
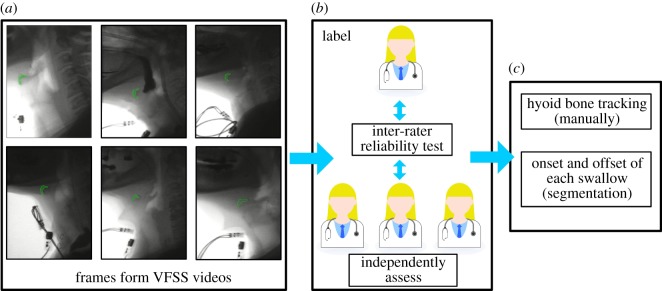
The dataset included 400 swallowing cases accompanied by hyoid bone tracking annotations used to train the SRNN. In (*a*), the green areas indicating the hyoid bone are approximated for the reader’s convenience. In (*b*), the hyoid bone location in each frame was manually labelled by one experienced rater and the inter-rater reliability test was implemented with three other frame raters. The swallowing segmentation was also labelled by one rater and the inter-rater reliability test was implemented with another rater, as shown in (*c*).

Human raters measured the duration of each swallow segment and marked the position of the hyoid bone body on each frame (*n* = 16 891) of each swallow in the dataset, as shown in figure 2. The height and width of each frame were 1008 pixels and 792 pixels, respectively. Each swallow was segmented by determining the beginning and end of each pharyngeal swallow in order to obtain individual swallows within a specific time duration. In the VFSS analysis, we defined the pharyngeal swallow segment as the duration between entry of the head of the bolus into the pharyngeal space, using the ramus of the mandible as an anatomical reference for the division between oral and pharyngeal cavities [2,25]. According to such a criterion, onset of a swallow segment was defined as the frame in which the leading edge of the bolus passed the radiographic shadow of the ramus of the mandible, and offset was the time when the hyoid returned to its lowest position at the end of the swallow following clearance of the bolus from the pharynx. The inter-rater reliability test was also taken by another rater with 21 swallows, and the inter-rater correlation coefficient was 0.998. To calculate such a coefficient value, we used the statistics software SPSS (v. 22) from IBM, in which the mixed effects model for absolute agreement and multiple raters was conducted [26].

**Figure 3.**
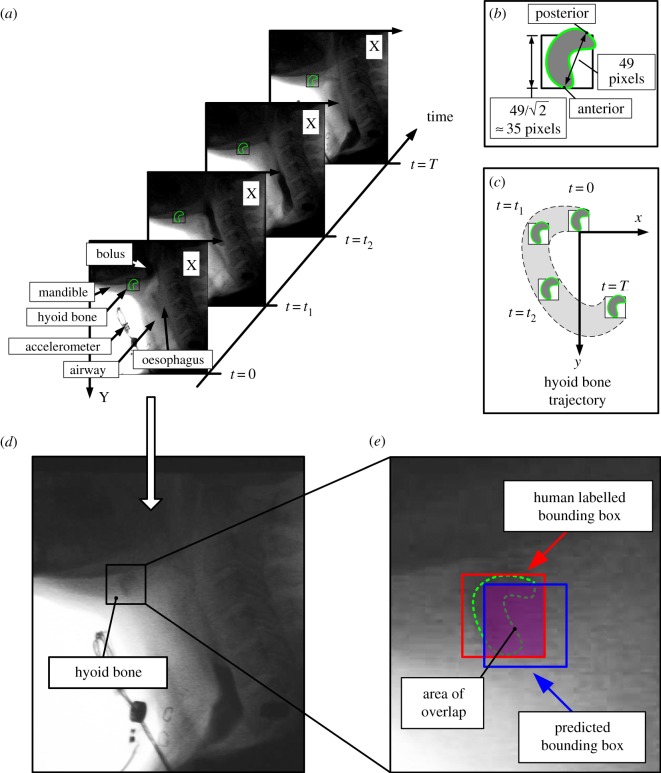
The hyoid bone movement for model training was determined from human raters of VFSS images and was measured as a two-dimensional time sequence corresponding to the *x* and *y* spatial axes, as shown in (*c*). The bounding box represented the hyoid bone as shown in (*b*). The hyoid bone’s demarcation line in green was approximated for reader’s visualization. However, owing to reduced image quality, the demarcation of the hyoid bone was often unclear, as shown in (*d*). The coordinates of the anterior and posterior points were used to locate the hyoid bone, as shown in (*b*). The anterior point of the hyoid bone was tracked in this study, because the posterior point was not always visible. In (*a*), when *t* = 0, *t*_1_ and *t*_2_, the posterior point was totally shaded. (*e*) The bounding boxes helped confirm the effective area of the hyoid bone and the overlapped area between the bounding boxes gives the accuracy in hyoid bone tracking.

To determine hyoid bone movement, a human rater marked the A–P landmark point of the body of the hyoid bone in each frame, as shown in figure 3*a*. One trained rater labelled all 400 swallowing samples. Owing to reduced image quality from VFSS images, it can be challenging even for human judges to accurately identify the outline of the hyoid bone during frame by frame analysis. To mitigate this problem, we drew a square bounding box to approximate the body of the hyoid bone. To determine the size of the bounding box, we found the average length between the anterior and posterior points of the hyoid bone, which was 49 pixels. Therefore, we used 49 pixels for the length of the diagonal of the bounding box and 35 pixels for the length, as shown in figure 3*b*. The labelled hyoid bone movement was further standardized in terms of each participant’s vertebral length, and to enable correction for patient movement during swallowing, for model training.

To evaluate the variation between human raters, a reliability test was implemented. We had three trained human raters complete frame-by-frame analysis of hyoid bone movement for 40 swallows (10% of the total samples) and then calculated the overlapping percentage:

2.1 $$\eta_{H-H}=\frac{\sum_{\;j=1}^{40}\sum_{t=1}^{M_{\;j}}\eta_{H-H}(t)}{\sum_{\;j=1}^{40}M_{\;j}}\times 100\text{\%}.$$

**Figure 4.**
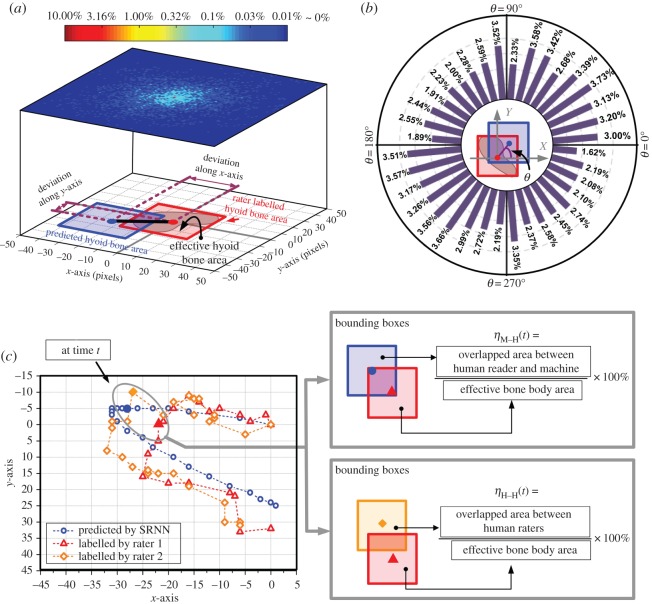
Hyoid bone movement tracking results of the *in silico* test. The blue and red bounding boxes denote the predicted and human rater labels of hyoid bone location, respectively. The distribution of the deviation is shown in (*a*), and the colour bar shows the proportions corresponding to different pixel coordinates. The angular deviation is also summarized in (*b*), which is presented in the histogram according to the variation of *θ*. In (*a*,*b*), we set the centre point of red boxes (human labelled hyoid bone) at the origin of coordinates for clear comparison. An exemplary result of hyoid bone tracking is shown in (*c*) with the comparison of two human raters’ annotated results.

The time-dependent coefficient $\eta_{H-H}(t)$ is defined in figure 4*c* (Results section), and *M*_*j*_ is the total time points of selected swallow *j*. The overall $\eta_{H-H}$ is a constant presenting the variation of the human-labelled hyoid bone trajectories.

### 2.3. Stacked recurrent neural network for deep learning

The SRNN structure was extended deeper by stacking multiple recurrent hidden layers (***h***) on top of each other. Such an architecture creates more efficient networks in means of deep transitions between consecutive hidden states [27–30]. The output sequence of each hidden layer ***h***_*k*_ is computed from input sequence ***h***_*k*−1_ through the following nonlinear relationship:

2.2 $$h_{k}^{\left(t\right)}=\left\{ \;\begin{array}{l} \sigma \;(W_{k}h_{k}^{\left(t-1\right)}+V_{k-1}h_{k-1}^{\left(t\right)}+b_{k-1})\;k=2,\ldots ,m\\ \sigma \;(W_{1}h_{1}^{\left(t-1\right)}+Rx^{\left(t\right)}+b_{0})k=1, \end{array}\right.$$

where *σ* is a nonlinear function which introduces the nonlinearities into the model. We selected rectified linear unit as *σ* . x(t) is the feature vector of the sensor signal. The final output $\hat{y}\left(t\right),$ which predicted the hyoid position at time *t*, was calculated from the last recurrence layer ($h_{m}^{\left(t\right)}$) with a linear combination, namely:

2.3 $$\hat{y}(t)=Uh_{m}^{\left(t\right)}+b_{m}.$$

In this study, the SRNN had four hidden layers and 64 neurons in each layer. At the start of model training, the recurrent weight matrix (***W***_*k*_) was initialized to a normalized-positive definite matrix with highest eigenvalue of unity and all the remainder eigenvalues less than 1 [27,28,31]. The input weight matrix ***R***, intermediate weight matrix (***V***_*k*_) and the output weight matrix (***U***) were randomly sampled in [− 0.01, 0.01]. All the biases (***b***) were initialized as zeros.

### 2.4. Data processing and feature extraction

The target sequence, which was the desired output of the SRNN, was generated from the marked hyoid bone location on VFSS images. For each frame, we first created a referential axis according to C2–C4 landmarks and calculated the hyoid bone position with this axis. Then we removed the offset of the swallow from the displacement sequence of the anterior point to determine the hyoid bone movement:

2.4 $$y_{\mathrm{Ant}-\mathrm{Traj}}(t)=y_{\mathrm{Ant}-\mathrm{Image}}(t)-y_{\mathrm{Ant}-\mathrm{Image}}(0).$$

Then, we scale ***y***_Ant −Traj_(*t*) to the range of [0, 1], namely:

2.5 $$y_{k}(t)=\frac{y_{\mathrm{Ant}-\mathrm{Traj},k}(t)-\min[y_{\mathrm{Ant}-\mathrm{Traj},1∼N}(t)]}{\max[y_{\mathrm{Ant}-\mathrm{Traj},1∼N}(t)]-\min[y_{\mathrm{Ant}-\mathrm{Traj},1∼N}(t)]}.$$

The subscript *k* is the swallow index and *N* is the total sample number of the training set, which is 280 and will be introduced later.

The input of the SRNN model was generated from the sensor signals, which were down sampled from 20 kHz to 4 kHz to remove the redundant points. Then, the displacement of the sensor was obtained by numerically double-integrating the signal using the trapezoid rule. This displacement was further down sampled to 30 Hz to match the sampling rate of VFSS. Meanwhile, according to the target (VFSS) time interval, which was 33 ms, the raw signals were separated into slices and each slice included 133 time steps. In each slice, we calculated the mean value and variance as additional features. For each swallow *k*, the input ***x*** was a time-dependent vector involving the sensor displacement, signal features in slices and a time variable *t*, namely:

2.6 $$\begin{array}{rl} x_{k}(t) & =[{\mathrm{Dis}}_{A-P}(t),{\mathrm{Dis}}_{S-I}(t),{\mathrm{Dis}}_{M-L}(t),{\mathrm{mean}}_{A-P}(t),{\mathrm{mean}}_{S-I}(t),{\mathrm{mean}}_{M-L}(t),\\ & \;{\mathrm{Var}}_{A-P}(t),{\mathrm{Var}}_{S-I}(t),{{\mathrm{Var}}_{M-L}(t),t]}^{T}. \end{array}$$

It is possible to let the SRNN discover any time-dependence when using *t* as an extra input [32]. The last step is the input normalization, which is calculated as:

2.7 $$x_{k,\mathrm{scaled}}(t)=\frac{x_{k}(t)-\min[x_{1∼N}(t)]}{\max[x_{1∼N}(t)]-\min[x_{1∼N}(t)]}.$$

The maximum and minimum values of both input and target were calculated from the training set. In the *in silico* test, the inputs of the testing samples were generated from the corresponding signal and applied equation (2.7) for normalization.

### 2.5. Model training and *in silico* test

For better generalization, a 10-fold cross-validation technique was used for the 400 swallowing samples, which were randomly divided into 10 non-overlapping groups. Each group was used as a testing set. The SRNN model was trained by nine other groups: 280 samples (70%) were used as the training set and 80 samples (20%) were used as the validation set. Samples collected from the same participant were assigned to a unique group. The model training process iteratively minimizes the error between model outputs and the targets of the training set. The hyoid bone movements of the training set determined and updated the weights using gradient descent with adaptive learning rate. The stop criterion was early stop, which indicates that the network was validated for minimum error on the validation set to avoid over-fitting. After the model was properly trained, all the parameters of SRNN were frozen, the training set was discarded from further inclusion in the analysis, and the samples in the testing set were used for evaluating the model performance.

In order to evaluate the accuracy of predicted hyoid bone movement, we first applied the proportion of overlapped area. The overlapped percentage in this paper is defined as follows:

2.8 $$\eta_{M-H}(t)=\frac{2\times \left(\mathrm{true}\;\mathrm{bounding}\;\mathrm{box}\;\mathrm{area}\right)\;\cap \;\left(\mathrm{predicted}\;\mathrm{bounding}\;\mathrm{box}\;\mathrm{area}\right)}{\left(\mathrm{true}\;\mathrm{bounding}\;\mathrm{box}\;\mathrm{area}\right)+\left(\mathrm{predicted}\;\mathrm{bounding}\;\mathrm{box}\;\mathrm{area}\right)}\times 100\text{\%}$$

2.9 $$\;=\frac{\text{area of overlap}\;(t)}{\text{area of bounding box}}\times 100\text{\%}=\frac{\left[D-|y_{x}(t)-{\hat{y}}_{x}(t)|]\times [D-|y_{y}(t)-{\hat{y}}_{y}(t)|\right]}{D^{2}}\times 100\text{\%}.$$

The overlapped area is provided in figures 3*e* and 4*c*. The red and blue bounding boxes present one human rater’s judgement and predicted hyoid bone body at time point *t*, respectively. In equation (2.8), the constant *D* is the side length of the square, which is 35 pixels as mentioned in figure 3. The subscript *x* and *y* for *y*(*t*) and $\hat{y}(t)$ are the hyoid bone coordinates on the *x*-axis and *y*-axis, respectively. The subscription ‘M–H’ indicates that the percentage is a comparison between the machine and one human rater.

In the training process, we applied early-stop strategy to avoid over-fitting, and force the SRNN to fetch the generalized pattern rather than the accurate positions of the anterior points. For the *in silico* test, it will be problematic to directly calculate overlapped percentage based on equation (2.8) owing to the variation between human raters. Considering the reliability test in labelling the hyoid bone movement, we can relax the constraints because of the variability between human raters by calculating the relative overlapped percentage (ROP):

2.10 $$\text{ROP}(t)=\frac{\eta_{M-H}(t)}{\eta_{H-H}}\times 100\text{\%}.$$

The ROP is essentially a comparison between the artificial intelligence and human rater (comparative test). When the ROP approaches 100%, it means that the RNN’s tracking output is nearly identical to human rater’s judgement, and when the ROP is, for example 50%, the RNN’s tracking output has detected at least 50% of the body of the hyoid bone in the measurement frame.

## 3. Results

This study aimed to track hyoid bone movement using signals acquired from sensors placed on the human neck, as shown in figure 1. The relationship between signals and hyoid bone movement was explored by SRNN using information from training samples. In order to track the hyoid bone movement of an unseen testing swallowing sample (*in silico* test), we used only signals from the accelerometry sensors. The parameters of the SRNN (weights and biases) were frozen at test, which means that the network’s evaluation behaviour was solely the result from information of training samples.

In the experiment, every swallowing sample was tested once since the 10-fold validation technique was implemented. We first quantified the tracking performance when the deviation is located at different distances (in pixels) and directions compared with one human rater, as shown in figure 4. To do this, we determined the predicted error from the SRNN prediction of the distance and direction of hyoid bone movement by examining the proportion distribution at all time points (frames) for all swallows. For all predicted points, 50.27% were located in the range of ±17 pixels (compared with one human rater labelled hyoid bone location), which is within the boundary box denoting the actual location of the hyoid bone. The angle deviation *θ*, which is defined in figure 4*b*, was approximately uniformly distributed in all directions.

**Figure 5.**
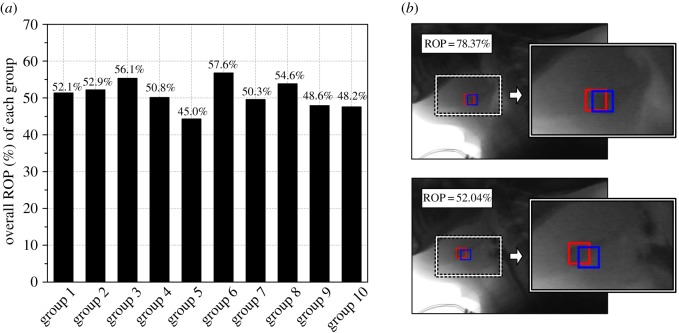
The average ROP of each group is shown in (*a*), two examples of different ROPs are shown in (*b*).

At each time point (frame), the SRNN predicted location of the hyoid bone centred mostly on the actual visually labelled hyoid bone location. However, it should be noted that the results in figure 4*a*,*b*, which compare computer predicted and labelled hyoid bone, are calculated based on only one human rater’s judgement. Variability exists even across multiple human raters, because of reduced quality VFSS images that make it challenging to determine the exact location of the hyoid bone during swallowing. A diagrammatic demonstration is shown in figure 4*c* and illustrates the variation between two human raters of hyoid bone movement during frame-by-frame analysis. Because variability exists between human ratings of hyoid bone movement, it is important to explore the impact on the final prediction using SRNN. To account for this variability, we used a metric of ROP, which involved a reliability test among four human raters. This test is similar to the overlapped percentage calculated in the right bottom part of figure 4*c*, and the average *η*_H−H_ (defined in 4*c*) indicating all measures from the human raters was 79.05%. The swallowing samples were divided into 10 groups, which were tested individually. The mean value of the ROP for each group of swallows is summarized in figure 5 and the overall mean value of ROP among all 10 groups was 51.60%. According to the individual variation of the participants, the average ROPs are summarized in table 1.

**Table 1.** Average ROP grouped by history of stroke and gender.

	without history of stroke	with history of stroke	overall
overall	52.71%	48.25%	51.61%
sample number	301	99	400
male	49.91%	44.25%	48.19%
sample number	164	71	235
female	56.09%	58.42%	56.48%
sample number	137	28	165

Table 1 shows that on average, the ROP of swallows from patients without a diagnosis of stroke was higher than patients who did have a stroke. This may indicate the greater variability of swallowing physiology following stroke which disrupts neuromuscular integrity. For patients who did not have stroke histories, the average ROP between genders was 6.18% (56.09%–49.91%), while it was 14.17% (58.42%–44.25%) for patients who did have a stroke. A test swallow with two exemplary frames is shown in figure 5*b*, in which the ROPs are 78.37% and 52.04%, respectively. All these results were identified by the sensor signals.

## 4. Discussion

The primary aim of this research study was to determine if computer prediction using SRNN could accurately detect hyoid bone movement during swallowing using signals from the accelerometer compared to human raters of hyoid bone movement. We demonstrated that a highly complex and nonlinear relationship between hyoid bone movement and sensor signals can be established via advanced machine learning algorithms such as SRNN. From our experimental results, the average tracking accuracy for hyoid bone movement from the SRNN highly approximated the human rater’s judgement, which provides preliminary evidence to support our hypothesis.

Previous studies have investigated the association between sensor signals and hyoid bone movement. These studies found that the signals had sufficient information to reliably estimate hyoid bone related movement during swallowing [17,19,20]. These studies examined the association between signals and hyoid bone movement in an individual participant first and then discussed the universal pattern among the cohort with a linear statistical model. However, to our knowledge to date, no research studies have attempted to quantitatively track real-time hyoid bone movement during swallowing. Therefore, this study aimed to expand upon this prior work by considering two properties of hyoid bone movement: the nonlinearity of hyoid movement, and the serial dependency between hyoid positions in adjacent video frames and, consequently, during swallowing. The relationship between signals acquired from the human neck and hyoid bone movement is mathematically nonlinear, because swallowing is an anatomically complicated neuromuscular process. Likewise, the relationship between signals and hyoid bone movement is time-dependent, meaning that hyoid bone location at any given point in time is influenced by where the hyoid bone has previously been during the swallow. Both properties were taken into account by the SRNN structure described in this research in order to track hyoid bone movement in real time to improve the clinical use of using non-invasive sensors to assess physiological components of swallowing.

Based on the results, the proposed deep learning method detects hyoid bone movement more accurately for patients who have not had a stroke than patients who have had a stroke based on the ROP. One possibility for this discrepancy between groups is that neurological damage following stroke may result in impaired hyoid bone movement, which could lead to more unpredictable movement patterns. This finding leads to intriguing research questions regarding the ability of sensor signals to distinguish characteristics of disease-specific patterns of swallowing disorders that may aid in differential diagnosis. In addition to this, we examined the signals in both men and women. Prior research studies have reported differences in some parameters of swallowing between men and women [33,34]. The structural features of the cricoid cartilage, the site of accelerometer placement, are different between males and females. However, based on our analysis, there were no significant differences between genders for tracking accuracy unless they had a history of stroke. For patients with history of stroke, there were significant differences in hyoid bone movement between genders. These results underscore that, as with human judgement, disease-related dysphagia poses more difficult analysis by both humans and sensor-based systems, though unlike human training, the signals continue to possess significant potential for algorithm refinement. In order to eliminate the extreme cases, such as severe post-stroke dysphagic swallowing, we excluded the multiple and sequential swallowing samples in the analysis. Further research work should determine if the sensor signals may be able to elucidate whether these differences in hyoid bone movement between groups exist owing to gender or other factors such as stroke severity, stroke location or dysphagia severity, because the abnormal hyoid bone movements should also affect the neck sensor signals and provide information. Future efforts may also investigate potential interaction effect of patient’s age and the variety of bolus volumes on the performance of hyoid bone tracking. However, because the aim of the study was to determine the ability of sensor to independently track the hyoid bone regardless of age, gender or diagnosis, these additional considerations have led to interesting directions for future research.

In the clinical setting, patients undergo screening to identify the likelihood of dysphagia, and then may undergo imaging studies to measure the actual components of swallowing dysfunction and generate timely interventions to mitigate the adverse effects of dysphagia. Without accurate screening, many patients’ dysphagia can go undetected, exposing patients to harm. Traditional screening methods for dysphagia are somewhat subjective and have limited accuracy, influenced mainly by poor specificity [1]. As a potential adjunct to screening, in which hyoid and other physiologic swallowing events are completely undetectable, a sensor-based technique offers a more objective way to identify the likely presence of disordered swallow function in patients with suspicious diagnoses or otherwise elevated risk. For example, this study investigated the ability of signals to perform tracking of hyoid bone movement, which is not measurable without VFSS, and which is strongly associated with airway closure and upper oesophagel sphincter opening. Detection of disordered hyoid bone movement with signals at the screening stage may help to detect physiological swallowing impairments earlier than is currently possible, to more accurately and quickly identify whether a patient has dysphagia and/or a high risk of aspiration before they are placed at risk of airway obstruction or other adverse consequences.

We double integrated the accelerometer signals in all three directions to obtain information about hyoid bone movement to use as inputs in the model. While it is common to use integration, it can result in more errors [35–37]. However, based on the results, these errors did not significantly affect the model’s ability to track hyoid bone movement. There are three explanations for this minor effect: (i) integration was implemented over a short time period (average swallow segment duration was 0.88 s); (ii) we down sampled the signal from 20 kHz to 4 kHz and removed device noise in order to make the signals more accurate and reliable; and (iii) the training iterations of the SRNN automatically extracted meaningful features from sensor signals. The impact of gravity on A–P hyoid bone movement was probably one source of bias in the model, but this can be corrected with time-dependent SRNN.

## 5. Conclusion

In this study, we proposed a new method for tracking hyoid bone movement based on neck sensor signals. Using deep SRNN, we examined the accuracy in tracking hyoid bone movement in real time using accelerometer signals compared to gold-standard human measurements. Results revealed that it is feasible and possible to track hyoid bone movement solely based on information provided from sensor signals. This study also found that the performance of hyoid bone movement tracking was influenced by patient diagnosis. This provides preliminary evidence for using the sensor as a non-invasive swallow screening instrument and tool to track hyoid bone movement. Further investigation of the sensor’s potential diagnostic value is warranted and currently underway.

## Supplementary Material

Average ROP grouped by history of stroke and gender

## Supplementary Material

A hyoid bone movement comparison sample

## Supplementary Material

Hyoid bone movement label

## Supplementary Material

The neck sensor signals

## Data accessibility

Data are included as the electronic supplementary material.
